# Cognitive impairment and associated factors among mature and older adults living in the community of Gondar town, Ethiopia, 2020

**DOI:** 10.1038/s41598-022-11735-2

**Published:** 2022-05-09

**Authors:** Yibeltal Yismaw Gela, Sofonias Addis Fekadu, Yitayeh Belsti, Yonas Akalu, Baye Dagnew, Mihret Getnet, Mohammed Abdu Seid, Mengistie Diress

**Affiliations:** 1 Department of Physiology, College of Medicine and Health Sciences, University of Gondar, Gondar, Ethiopia; 2 Department of Optometry, College of Medicine and Health Sciences, University of Gondar, Gondar, Ethiopia; 3 Unit of Physiology, Biomedical Department, College of Medicine and Health Sciences, Debre Tabor University, Debre Tabor, Ethiopia

**Keywords:** Neuroscience, Physiology, Medical research, Neurology

## Abstract

Cognitive impairment and dementia are age-related major public health concerns in the elderly population. It is a major cause of disability, dependency, and poor quality of life. However, in Ethiopia, the magnitude of this cognitive impairment among the elderly community was not investigated. Hence, this study sought to determine the prevalence of cognitive impairment and associated factors among mature and older adults living in the community of Gondar town, Ethiopia, in 2020. A community-based cross-sectional study was conducted at Gondar town, from February 20 to April 30, 2020. Using a single-stage cluster sampling technique, 403 study participants were recruited. Data was collected by a pretested interviewer-administered structured questionnaire which consisting of sociodemographic variables, the Oslo Social Support Scale, and a Standardized Mini-mental State Examination (SMMSE) tool. Epi data version 3.0 was used to enter coded data and then exported into STATA 14 for analysis. Variables with a p-value < 0.25 in the bi-variable logistic regression were included in the multivariable regression. From multivariable logistic regression, variables having a p-value ≤ 0.05 were declared as statistically significant variables. In this study, a total of 403 study participants were involved, and 393 (97.5%) of them completed the survey. Among older participants screened for cognitive impairment, 43.8% was positive for cognitive impairment with a 95% CI (38.8–48.7%). The majority of the participants were (57.5%) male and (44.8%) aged over 60 years. After adjustment, the variables associated with cognitive impairment were age ≥ 75 years [odds ratio (OR) = 7.03, 95% CI 2.78–17.77] and between 61 and 74 years [OR = 3.18, 95% CI 1.81–5.59], and unable to read and write [OR = 5.05, 95% CI 2.04–12.50], low income level [OR = 2.60, 95% CI 1.26–5.20], being female [OR = 2.52, 95% CI 1.50–4.26], poor social support [OR = 2.50, 95% CI 1.30–4.81], and rural residence [OR = 2.39, 95% CI 1.26–4.51]. The prevalence of older participants who screened positively for cognitive impairment was high at Gondar town. The independent predictors of cognitive impairment among older individuals were older age, being unable to read and write, being female, low income, poor social support, and rural dwelling. Therefore, routine screening and social support, as well as free healthcare services for the mature and older community, are needed. Moreover, we strongly recommend the next researcher to use a diagnosis tool to estimate the actual prevalence of the problems among older people.

## Introduction

Cognitive impairment is defined as difficulty recalling, learning new things, focusing, or making decisions that affect one’s daily life^1^. It affects the orientation, attention, reasoning, memory, language, and executive functions of individuals^2^. Those affected elderly people had poor concentration, a short attention span, emotional disturbance^3^, and impairment of recent memory^4,5^.

Cognitive impairment and dementia are common age-related health problems among older people^6^. The proportion of older people is increasing in every country. By 2050, around two billion people worldwide will be aged 60 years or over^7^. Currently, around 50 million people live with a severe form of cognitive impairment worldwide, 60% of whom are from developing countries. Every year, around 10 million people are diagnosed with dementia^8,9^. The prevalence of severe cognitive impairment is expected to be 82 million in 2030 and 152 million by 2050^8,10^. The problem is increasing worldwide and predicted to increase consistently more in developing countries^11^.

According to the 2007 census, 9% of the population in Ethiopia was aged ≥ 50 years, which is estimated to increase progressively^12^. Currently, family caregivers for elderly relatives in Ethiopia are generally poor^13^ and only half a million older people receive regular public sector pensions^14^.

The prevalence of cognitive impairment among older individuals was (42%) in French^15^, (28.5%) in Congo^11^, (37.9%) in the Central African Republic^16^, (20.9%) in Nigeria^17^, and (33.3%) in Cameroon^6^.

Cognitive impairments have health, psychological, social, and economic impacts. Its impact on health^3,5,6^ was increased rates of hospitalization, disability, and risk of falls. In addition, individuals with cognitive impairment have an increased risk of death, poor quality of life, worse quality of emotional well-being, and longer hospitalizations^3,18,19^. Severe cognitive impairment is now the seventh leading cause of death in all diseases^20^. Severe cognitive impairment deaths in Ethiopia reached 8316, or 1.36% of total deaths. The age-adjusted death rate is 24.48 per 100,000 population, which ranks Ethiopia 92th in the world^21^.

Stigma, dependency, and discrimination are also common psychological and social problems among people with severe cognitive impairment^10,22^. Severe cognitive impairments have an impact not only on the people living with severe cognitive impairment, but also on their caregivers, families, and society at large^20^. Informal caregivers, such as family members and friends, spend an average of five hours per day caring for people with dementia. These physical, emotional, and financial pressures can cause great stress to families and caregivers^22^.

According to the World Health Organization, the annual cost of caring for people with severe cognitive impairments will reach two trillion dollars by 2030^8,9,22^.

Around 90% of people with severe cognitive impairment in low and middle-income countries do not obtain a diagnosis for this disorder^9^. In Ethiopia, cognitive impairment is expected to increase as the life expectancy of the population increases. Even though the burden of this health problem is huge, the prevalence and associated factors of cognitive impairment were not investigated among older communities in Ethiopia. Therefore, this study is aimed at investigating the prevalence of cognitive impairment and associated factors among mature and older adults living in the community of Gondar, Ethiopia, in 2020.

## Methods and materials

### Study area and period

A community-based cross-sectional study was conducted in Gondar town, from February 20 to April 30, 2020. Gondar town is located 750 km away from Addis Ababa (the capital city of Ethiopia) in the north-west direction. It is one of the ancient and densely populated towns in Ethiopia. According to a 2007 Ethiopian Central Statistical Agency office report, around 206,987 people are living in the town^23^.

### Study population

All older people aged 50 years or above were included in this study.

#### Inclusion and exclusion criteria

All adults aged 50 years or older whose residence was in the selected kebeles were included in the study.

In this study, all elderly participants with hearing, visual, and speaking difficulties were excluded from the study.

### Sample size determination

The sample size was estimated using a single population proportion formula by assuming a 50% prevalence of cognitive impairment, since there was no study conducted before in Ethiopia, a 95% confidence interval, and a 5% marginal error.

$$\mathrm{N}= \left[\frac{{ \left({\mathrm{Z}}_{\alpha/2}\right)}^{2} \times \mathrm{p}\left(1-\mathrm{p}\right) }{{\mathrm{d}}^{2}}\right]=\frac{{\left(1.96\right)}^{2} \times 0.5(1-0.5) }{{(0.05)}^{2}}=384$$

N = sample size, Z_α/2_ (1.96) = critical value at 95% confidence interval, p = expected estimates of prevalence value of cognitive impairment (50%), d = Margin of sampling error (5%). After adding 5% of the non-response rate, a total of 403 participants were selected.

### Sampling procedures

A single-stage cluster sampling technique was employed to recruit the study participants. In Gondar town, there are 22 kebeles, which we considered as a cluster. From those 22 kebeles, (kebele 6, kebele 7, kebele 8, kebele 9, kebele 13, kebele 15, kebele 16, kebele 17, kebele 18, kebele 20) were randomly selected by using the lottery method. Then all adults with aged 50 years or above whose residences were in those selected kebeles were included in the study.

This study was conducted under the principle of the Helsinki Declaration. Ethical clearance was obtained from the Institutional Review Board of the University of Gondar. During the data collection period, the aim of the study was explained for each participant. Written informed consent was obtained from each study participant before we started the data collection process. The privacy and confidentiality of participant information were also kept properly.

### Study variables

*Dependent variable* cognitive impairment (yes/no).

Independent variables;

*Socio-demographic variables* (age, sex, residence, social support, monthly income, marital status, and educational status).

*Lifestyle and medical history related variables*: substance use (khat chewing, cigarette smoking, and alcohol intake), overweight or underweight.

### Operational definitions

In our study, mature and older adult was defined as being 50 years of age or above^6^.

#### Cognitive impairment

Using the Standardized Mini Mental State Examination (SMMSE) tool, participants with an educational level of ≤ grade 8 with scores of ≤ 22 and participants with an educational level of ≥ grade 9 with scores of ≤ 24 out of a total of 30 scores , had cognitive impairment^24,25^.

The stage of cognitive impairment was classified as a score of 20–24 as mild cognitive impairment, a score of 10–19 as moderate cognitive impairment, and a score of 0–9 as severe cognitive impairment for participants with ≥ 9 grade, and a score of 18–22 as mild, 8–17 as moderate, and 0–7 as severe cognitive impairment for participants with ≤ 8 grade^24,25^.

#### Social support

Using the Oslo Social Support Scale, participants who scored 3–8, 9–11, 12–14 points out of a total score had poor social support, moderate social support, and strong social support, respectively^26^.

#### Substance use

Khat chewers or cigarette smokers are those participants who had used khat or cigarettes in the previous month, while those individuals who had consumed alcohol that could cause intoxication in the previous month were alcohol drinkers^27,28^.

#### Body mass index (BMI)

Participants with BMI of < 18.5 kg/m^2^, (18.5–24.9 kg/m^2^), (25–29.9 kg/m^2^), ≥ 30 kg/m^2^ were classified as underweight, normal, overweight and obese, respectively^27^.

### Data collection procedure and tools

An interviewer-administered structured questionnaire, which consists of socio-demographic variables, substance use, weight and height measurement, the Oslo Social Support Scale (OSSS), and a standardized mini mental state examination tool (SMMSE tool), was used to collect the data^24,29^. House to house survey was undertaken to collect the data. A SMMSE is a validated tool that is used for screening the cognitive status of the participants. The mean duration for assessments of the cognitive status of participants by SMMSE was 11 min. The tools consist of questions that evaluate orientation, attention, orientation, registration, calculation, recall, language, and praxis parts of cognition, which result in a total of 30 scores when summed. The total score for each participant was adjusted according to the educational level of the participants^24^. The inability to read and write was taken into account when scoring the test for those participants who had omitted items due to being unable to read and write. The score from this task was subtracted from the total score of 30 to give a new total. The person’s score was then adjusted to the new total score^30^.

The Oslo Social Support Scale (OSSS) tool was used to assess the social support level of study participants. It consists of three items scored out of 14; one item (4 points) and the remaining two items (5 points each)^26^.

Moreover, weight and height were measured using a weighing machine and a height-measuring stand to the nearest 0.1 cm and 0.1 kg, respectively. The scale was adjusted to a zero level between individual measurements.

### Data processing and analysis

After the data have been checked for its completeness and coded, it is entered into Epi Data Version 3.0 and exported to STATA 14 for analysis. For categorical variables, descriptive statistics such as frequency, percentage, and bar graphs were used, whereas mean and standard deviation were used for continuous variables. Bi-variable and multivariable logistic regression analyses were done. In bi-variable logistic regression, variables associated with cognitive impairment at p-value < 0.25 were included in the multivariable regression model. Lastly, using a 95% confidence interval, variables having a p-value ≤ 0.05 in multivariable logistic regression were confirmed as significantly associated with cognitive impairment.

Model fitness was checked by the Hosmer and Lemeshow goodness of test (at p > 0.05) and multi-collinearity was also tested by variance inflation factor (VIF). Moreover, a commonly used cognitive impairment screening tool (SMMSE) was used to assess the cognitive status of the participants with a sensitivity and specificity of 81% and 94%, respectively^31^. The instrument's reliability was also established based on an internal consistency reliability assessment using Cronbach’s alpha (= 0.8), indicating that the tool is reliable.

### Data quality assurance

To assure the quality of the data, training was given by the principal investigator to the data collectors with regard to the SMMSE tool and the procedure for height and weight measurement. For the consistency of the questionnaire, after it was translated to Amharic language by a language expert again retranslated back to English language by another expert. A pretest was conducted among 40 participants in Bahirdar town, then the questionnaire was amended accordingly. The supervisor and principal investigator supervised the data collectors during the data collection period. Protective materials and physical distance were applied by the data collectors during data collection for the prevention of COVID-19 transmission.

## Results

### Sociodemographic characteristics of study participants

In this study, a total of 393 participants were involved, with a 97.5% response rate. The mean age of study participants was 63.6 years and 50 and 92 years were the minimum and maximum ages, respectively. Most of the study participants were male (66%), orthodox Christian followers (86.2%), attended primary school or less (42%), married (82%), and employed (34.9%). In this study, 5 (1.3%), 63 (16%), and 2 (0.5%) of the participants were khat chewers, alcohol users, and cigarette smokers, respectively. Those substance users used at least half a gram of khat and one stick of cigarate per month, and 2–3 L of alcohol per week (Table 1).

**Table 1:** Socio-demographic characteristic of study participants on cognitive impairment and associated factors among elders at Gondar town, 2020.

Variables		Number (%)
Sex	Male	226 (57.5)
	Female	167 (42.5)
Age (years)	50–60	175 (44.5)
	61–74	176 (44.8)
	≥ 75	42 (10.7)
Religion	Orthodox	323 (82)
	Muslim	50 (13)
	Protestant	20 (5)
Educational level	Unable to read and write	85 (21.6)
	Grade 1–8	165 (42)
	Grade 9–12	67 (17.1)
	College and above	76 (19.3)
Marital status	Married	368 (94)
	Divorced	16 (4)
	Widowed	9 (2)
Occupation	Employed	215 (54.7)
	Merchant	69 (17.6)
	Farmer	54 (13.7)
	Housewife	55 (14)
Income (ETB)	≤ 1500	77 (19.6)
	1501–3500	105 (26.7)
	≥ 3501	211 (53.7)
Residence	Urban	296 (75.3)
	Rural	97 (24.7)
BMI (kg/m^2^)	Normal	333 (84.7)
	Underweight	28 (7.1)
	Overweight	32 (8.2)
Social support	Poor social support	103 (26.2)
	Moderate support	122 (31)
	Strong support	168 (42.8)

### Prevalence of cognitive impairment among elderly community

The mean standardized mini-mental state examination score of the study participants was 23.3 (standard deviation ± 0.24).

**Figure 1:**
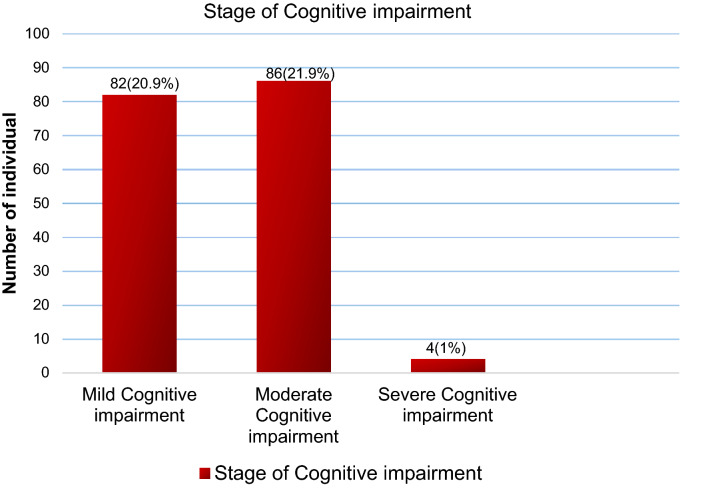
Stage of cognitive impairment among mature and older adults living in the community of Gondar town, Ethiopia, 2020.

Among older participants screened for cognitive impairment, 43.8% was positive for cognitive impairment with a 95% CI (38.8–48.7%). Most of them, 21.9% had moderate cognitive impairment (Fig. 1).

### Factors associated with cognitive impairment in the elderly population

In binary logistic regression, age, sex, educational level, income level, social support, and body mass index were associated with cognitive impairment at a p-value < 0.25. However, in the multivariable logistic regression analysis, low educational level, female sex, poor social support, rural residence, low income, and older age were variables significantly associated with cognitive impairment at p-value ≤ 0.05. The odds of developing cognitive impairment among the elderly community with an age of ≥ 75 years was 7.03 times [AOR = 7.03, 95% CI 2.78–17.77] higher than those individuals with an age between 50–60 years. The odds of developing cognitive impairment among elderly females was 2.52 times [AOR = 2.52, 95% CI 1.5–4.26] higher than males. The odds of developing cognitive impairment among elderly individuals who had poor social support was 2.50 times [AOR = 2.50, 95% CI 1.30–4.80] higher than those individuals who had strong social support. The odds of developing cognitive impairment among rural residents was 2.4 times [AOR = 2.4, 95% CI 1.26–4.51] higher than those of urban residents. The odds of developing cognitive impairment among the elderly participants with an income level of ≤ 1500 ETB was 2.6 times [AOR = 2.6, 95% CI 1.26–5.20] higher than those participants with an income level of ≥ 3501 Ethiopian birr (Table 2).

**Table 2:** Factors associated with cognitive impairment in binary and multiple logistic regression analyses among elderly community at Gondar town, 2020.

Variables		Total N (%)	Cognitive impairment		OR (95% CI)	
			Yes N (%)	No N (%)	COR	AOR
Age (year)	50–60	175 (44.5)	42 (24)	133 (76)	1	1
	61–74	176 (44.8)	99 (57.6)	77 (34.8)	4.07 ( 2.58–6.43)	3.18 (1.81–5.59)*
	≥ 75	42 (10.7)	33 (73.8)	11 (26.2)	8.92 (4.13–19.28)	7.03 ( 2.78–17.77)*
Residence	Urban	296 (75.32)	106 (26.7)	190 (73.3)	1	1
	Rural	97 (24.68)	66 (52.5)	31 (47.5)	3.82 (2.34–6.22)	2.39 (1.26–4.51)*
Sex	Male	226 (57.51)	76 (48.1)	150 (51.9)	1	1
	Female	167 (42.49)	96 (58.8)	71 (41.2)	2.67 (1.77–4.03)	2.52 (1.50–4.26)*
Education level	Unable to read and write	85 (21.6)	19 (22.3)	66 (77.7)	13.03 (6.14–27.61)	5.05 (2.04–12.50)*
	Grade 1–8	165 (42)	77 (46.7)	88 (53.3)	3.28 (1.75- 6.16)	1.55 (0.72–3.39)
	Grade 9–12	67 (17.1)	13 (19.4)	54 ( 80.6)	0.92 (0.40–2.05)	0.50 (0.19–1.33)
	College and above	76 (19.3)	16 (21.1)	60 (78.9)	1	1
Social support	Poor	103 (26.2)	60 (58.2)	43 (41.8)	3.5 (2.03–5.66)	2.5 (1.30–4.81)*
	Moderate	122 (31)	69 (51.6)	53 (48.4)	2.6 (1.59–4.22)	1.7 (0.93–3.09)
	Strong	168 (42.8)	49 (29.2)	119 (70.8)	1	1
Income level (ETB)	≤ 1500	77 (19.6)	52 (67.5)	25 (32.5)	3.69 (2.12–6.43)	2.6 (1.26–5.20)*
	1501–3500	105 (26.7)	44 (41.9)	61 (58.1)	1.3 (0.79–2.07)	1.2 (0.62–2.13)
	≥ 3501	211 (53.7)	76 (36.0)	135 (64)	1	1
BMI (kg)	Normal	333 (84.7)	150 (45)	183 (55)	1	1
	Underweight	28 (7.2)	15 (53.6)	13 (46.4)	1.41 (0.65–3.05)	1.38 (0.48–4.00)
	Overweight	32 (8.1)	7 (21.9)	25 (78.1)	0.34 (0.14–0.81)	0.38 (0.13–1.10)
Marital status	Divorced	16 (4.1)	8 (50)	8 (50)	1.34 (0.49–3.65)	2.9 (0.87- 9.69)
	Married	368 (93.6)	157 (42.7)	211 (57.3)	1	1
	Widowed	9 (2.3)	7 (77.8)	2 (22.2)	4.7 (0.96–22.95)	5.13 (0.88–30.07)

*P-value ≤ 0.05, COR: crude odds ratio; AOR: adjusted odds ratio; CI: confidence interval; N: number; ETB: Ethiopian Birr, BMI: Body Mass Index.

## Discussion

This research aimed to determine the magnitude of cognitive impairment and associated factors among mature and older adults living in the community of Gondar town.

Among mature and adult older participants screened for cognitive impairment, 43.8% was positive for cognitive impairment with a 95% CI (38.8–48.7%) which was higher than the studies done in Cameroon (33.3%)^6^, the Central Africa Republic (37.9%)^16^, Nigeria (19.7%)^17^, India (14%)^32^ and the Republic of Congo (28.5%)^16^. This variation might be due to the tool used to assess cognitive impairment. Community screening interview for dementia tool was used for Central Africa republic and Republic of Congo studies. In a study done in Nigeria, intervention for dementia in elderly Africans with cognition screening tools was used. It might also be due to the socio-demographic difference of study participants, in which in those studies, the participants had attained higher education.

As expected, different factors were capable of predicting cognitive impairment. In this study, getting older was associated with cognitive decline. This finding is in line with studies done in Cameroon^6^, Jamaica^5^, and Ethiopia^27^. The reason might be that as aging increase there is a decrement in neurotransmitters, gray matter volume, and neocortical synapses that result in cognitive impairment^33–35^. During aging the cerebrovascular reactivity is impaired, which result in brain hypoperfusion^36^. In addition to this, as age increases, there is deterioration in thinking, reasoning, and memory which is related to cognitive decline^37,38^.

Individuals who were unable to read and write were more likely to develop cognitive impairment than individuals attaining college and above, which is supported by other studies^5,39,40^. This might be due to education improving cognitive function through providing knowledge, understanding, skills, and experience^39,40^. It also promote cognitive function by increasing the number of synapse, vascularization, and promote neural development^39,41^.

In support of other studies^6,41^, low income level was significantly associated with cognitive impairment. It might be due to the inability to afford health care services for those participants with low income. Being female was associated with higher odds of developing cognitive impairment, which is similar to the finding of other studies^6,11^. This might be because of estrogen, progesterone, and testosterone hormones decline in women during menopause. Studies have shown that those hormones have a protective effect on the brain. They also reduce the level of amyloid beta peptide, which is negatively acting on the hippocampus^6,42^.

The odds of developing cognitive impairment among elderly individuals who had poor social support was higher than those individuals who had strong social support. This coincides with the studies conducted in Spain^43^ and Japan^44^. Social support influences both physical and mental health. Hence, those individuals with poor social support are more likely to develop cognitive impairment. It is hypothesized that poor social support associated with stress leads to corticosterone hypersecretion, which in turn leads to permanent loss of hippocampal neurons^45^.

Lastly, those individuals who are rural dwellers have a higher probability of developing cognitive impairment than elderly urban dwellers. This result is similar to studies conducted in China^46^ and India^47^. This might be due to an increase in educational and income levels among urban residents, which may lead to increased health-seeking behavior.

The findings of this research imply that cognitive impairment is becoming one of the health problems among adult and mature older community in Ethiopia.

## Limitations of the study

A SMMSE is a screening tool that cannot be used for the diagnosis of the outcome variable. Therefore, those participants might need further evaluation to confirm the problem. Moreover, since this study used a cross-sectional study design, it doesn’t show a cause and effect relationship.

## Conclusions

The prevalence of older participants who screened positively for cognitive impairment was high at Gondar town. The independent predictors of cognitive impairment among older individuals were older age, being unable to read and write, being female, low income, poor social support, and rural dwelling. Therefore, routine screening and social support, as well as free healthcare services for the mature and older community, are needed. Moreover, we strongly recommend the next researcher to use a diagnosis tool to estimate the actual prevalence of the problems among older people.

## Data Availability

The data will be available upon request from the corresponding author.

## Abbreviations

CI: Confidence interval
SMMSE: Standardized Mini Mental State Examination
